# 418. Low Frequency of Healthcare Worker Infections Following Occupational Exposures to COVID-19

**DOI:** 10.1093/ofid/ofab466.618

**Published:** 2021-12-04

**Authors:** Jessica Seidelman, Ibukunoluwa Akinboyo, Maya Rinehart, Matthew Stiegel, Rebekah W Moehring, Deverick J Anderson, Kristen Said, Carol A Epling, Sarah S Lewis, Becky Smith

**Affiliations:** 1 Duke University, Durham, NC; 2 Duke University Health System, Durham, North Carolina; 3 Duke Center for Antimicrobial Stewardship and Infection Prevention, Durham, NC; 4 Duke University Medical Center, Durham, North Carolina

## Abstract

**Background:**

Data on occupational acquisition of COVID-19 in healthcare settings are limited. Contact tracing efforts are high resource investments.

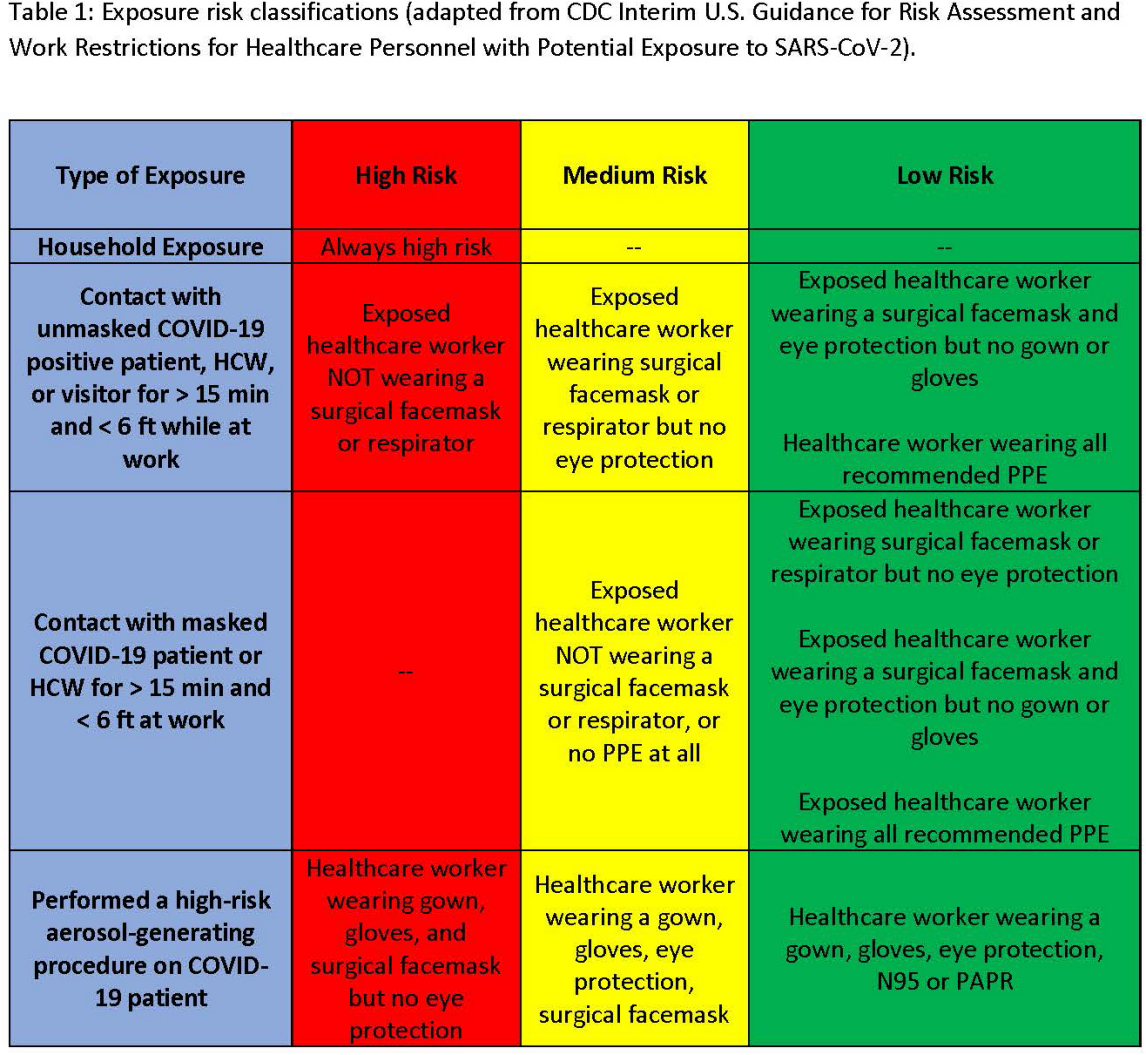

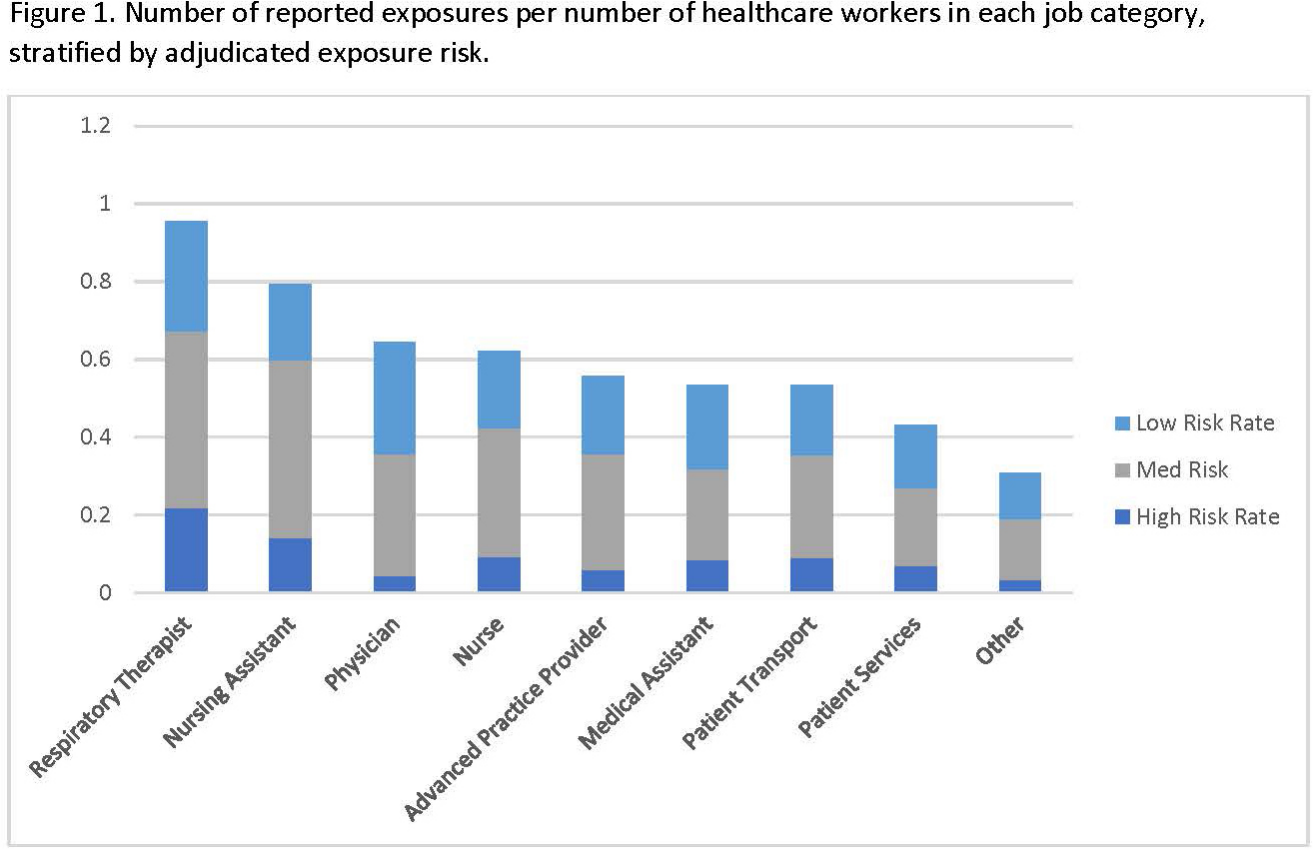

**Methods:**

Duke Health developed robust COVID-19 contact tracing methods as part of a comprehensive prevention program. We prospectively collected data on HCW exposures and monitored for development of symptomatic (SYX) and asymptomatic (ASYX) COVID-19 infection after documented high-, medium, and low-risk exposures. HCWs were required to self-report exposures or were identified through contact tracing as potentially exposed to COVID-19 positive HCWs, patients or visitors. Contact tracers interviewed exposed HCWs and assessed the risk of exposure as high-, medium-, or low-risk based on CDC guidance (Table 1). Testing was recommended at 6 days after high- or medium-risk exposures and was provided upon HCW request following low-risk exposures. Our vaccination campaign began in 12/2020.

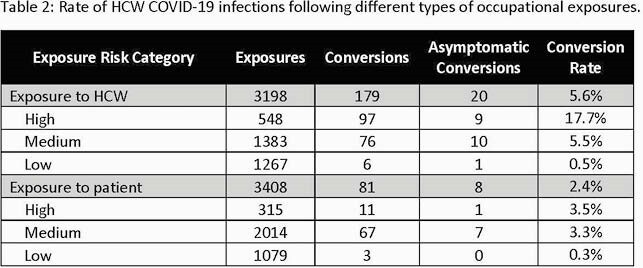

**Results:**

12,916 HCWs registered in the contact tracing database. From March 2020-May 2021, we identified 6,606 occupational exposures (0.51 exposures/HCW). The highest incidence of workplace exposures per number of HCWs in each job category was among respiratory therapists (RT) (0.95 exposures/RT), nursing assistants (NA) (0.79 exposures/NA), and physicians (0.64 exposures/physician). The most common exposure risk level was medium (51.4%), followed by low (35.5%), and then high (13.1%). A total of 260 (2%) HCW had positive tests/conversions; 28 (10.8%) were ASYX at the time of testing. High-risk exposures had a significantly greater number of post-exposure infections compared to medium- and low-risk exposures (12.5% vs. 4.2%, vs. 0.4%; p < 0.001). The rate of SYX infection following exposure to a fellow HCW (179/3,198; 5.6%) was higher than that following exposure to a patient (81/3,408; 2.4%; p< 0.001).

**Conclusion:**

Conversion following exposure to COVID-19 in the healthcare setting with appropriate protective equipment was low. Incomplete testing of all exposed individuals was a limitation and our data may under-estimate the true conversion rate. Our findings support our local practice of not quarantining HCWs following non-household exposures. Limiting contact tracing to only high or medium risk exposures may best utilize limited personnel resources.

**Disclosures:**

**Rebekah W. Moehring, MD, MPH**, **UpToDate, Inc.** (Other Financial or Material Support, Author Royalties)

